# Development of a rapid recombinase polymerase amplification assay for the detection of *Streptococcus pneumoniae* in whole blood

**DOI:** 10.1186/s12879-015-1212-5

**Published:** 2015-10-29

**Authors:** Eoin Clancy, Owen Higgins, Matthew S. Forrest, Teck Wee Boo, Martin Cormican, Thomas Barry, Olaf Piepenburg, Terry J. Smith

**Affiliations:** Molecular Diagnostics Research Group, School of Natural Sciences, National University of Ireland, Galway, Ireland; Biomedical Diagnostics Institute Programme, National Centre for Biomedical Engineering Science, National University of Ireland, Galway, Ireland; TwistDx Limited, Cambridge, United Kingdom; School of Medicine, National University of Ireland , Galway, Ireland; Nucleic Acids Diagnostics Research Laboratory, Microbiology, School of Natural Sciences, National University of Ireland, Galway, Ireland

**Keywords:** *Streptococcus pneumoniae*, Recombinase Polymerase Amplification, Leader peptidase A, Molecular diagnostics

## Abstract

**Background:**

*Streptococcus pneumoniae* is an important cause of microbial disease in humans. The introduction of multivalent vaccines has coincided with a dramatic decrease in the number of pneumococcal-related deaths. In spite of this, at a global level, pneumococcal infection remains an important cause of death among children under 5 years of age and in adults 65 years of age or older. In order to properly manage patients and control the spread of infection, a rapid and highly sensitive diagnostic method is needed for routine implementation, especially in resource-limited regions where pneumococcal disease is most prevalent.

**Methods:**

Using the gene encoding leader peptidase A as a molecular diagnostics target, a real-time RPA assay was designed and optimised for the detection of *S. pneumoniae* in whole blood. The performance of the assay was compared to real-time PCR in terms of its analytical limit of detection and specificity. The inhibitory effect of human genomic DNA on amplification was investigated. The potential clinical utility of the assay was investigated using a small number of clinical samples.

**Results:**

The RPA assay has a limit of detection equivalent to PCR (4.0 and 5.1 genome equivalents per reaction, respectively) and was capable of detecting the equivalent of <1 colony forming unit of *S. pneumoniae* when spiked into human whole blood. The RPA assay was 100 % inclusive (38/38 laboratory reference strains and 19/19 invasive clinical isolates) and 100 % exclusive; differentiating strains of *S. pneumoniae* species from other viridans group streptococci, including *S. pseudopneumoniae*. When applied to the analysis of a small number (*n* = 11) of clinical samples (blood culture positive for *S. pneumoniae*), the RPA assay was demonstrated to be both rapid and sensitive.

**Conclusions:**

The RPA assay developed in this work is shown to be as sensitive and as specific as PCR. In terms of reaction kinetics, the RPA assay is shown to exceed those of the PCR, with the RPA running to completion in 20 minutes and capable generating a positive signal in as little as 6 minutes. This work represents a potentially suitable assay for application in point-of-care settings.

## Background

*Streptococcus pneumoniae* is a major cause of human disease. It is estimated that more than 1.6 million people, including more than 800,000 children under the age of 5, die every year from pneumococcal infections [1]. In addition to its role as a leading cause of community-acquired pneumonia, *S. pneumoniae* is a causative agent of a variety of other infections, including otitis media, blood stream infection, spontaneous peritonitis and meningitis [2]. Pneumococcal meningitis is a form of pneumococcal disease associated with significant mortality and morbidity in children. In developed countries, pneumococcal meningitis has a mortality rate of 17–30 % [3] and an associated risk of sequelae of 32 % [4]. In developing countries, the mortality and morbidity rates are much higher [5]. *S. pneumoniae* belongs to the mitis subgroup of alpha-haemolytic (viridans) streptococci (VGS). Mitis group streptococci other than *S. pneumoniae* are also associated with bacteraemia which may be clinically non-significant but may also be associated with invasive disease, particularly in predisposed patients such as the immunocompromised and those with cardiac valve damage [6]. The mitis group organisms, *S. mitis*, *S. oralis* and *S. pseudopneumoniae*, possess >99 % 16S rRNA sequence homology with *S. pneumoniae* [7]. Furthermore, horizontal gene transfer between streptococci can give rise to atypical streptococci that are difficult to classify using phenotypic methods [8]. Given the close genotypic and phenotypic relationship between mitis group streptococci, the differentiation of pneumococcus from other VGS is challenging [9].

Culture based methods are the gold standard for diagnosing invasive pneumococcal disease (IPD). However culture based methods typically require at least several hours to a day or more incubation before an organism is detected. Furthermore, these methods often demonstrate limited diagnostic sensitivity, particularly post antibiotic treatment or in cases with low sample volume, as is often the case with children [10, 11]. Molecular amplification methods such as PCR and Loop-mediated isothermal amplification (LAMP) have been used to diagnose IPD [12, 13] . These methods are reported as sensitive, are not reliant on having viable organisms and have the potential to deliver more rapid results.

Recombinase polymerase amplification (RPA) is an isothermal *in vitro* nucleic acid amplification technology that uses a combination of recombinase protein, oligonucleotide primers and a strand displacing polymerase to amplify DNA sequences [14]. RPA is sensitive, rapid, specific and robust (less sensitive to inhibitors than PCR). RPA has been applied to the detection of a wide variety of bacterial, viral and eukaryotic molecular targets [15–22]. The use of a fluorescent probe enables monitoring of reactions in real-time and multiplexing capabilities. Isothermal amplification strategies are of great interest in molecular diagnostics, as they negate the need for thermocycling, as is the case for PCR. The isothermal nature of these techniques makes them suitable for incorporation into devices capable of being deployed at the point-of-care.

Molecular targets that have been used to identify *S. pneumoniae* include a variety of genes, including the Spn9802 fragment [23], the *RecA* gene [24] the 16S rRNA gene [25], and virulence factor genes, such as autolysin (*lytA*) [26] and pneumolysin (*ply*) [27, 28]. Whilst these targets have proven useful for the detection of *S. pneumoniae*, their ability to unequivocally identify *S. pneumoniae* remains problematic. For example, false positives can occur with the *lytA* marker [29] and both *ply* [30] and Spn9802 [23] are associated with false negative results. Leader peptidase A (*LepA*), also known as Elongation Factor 4 (EF4), is one of the most conserved bacterial proteins and is found in virtually all know genomes [31]. It functions as a ribosome dependant GTPase, with the ability to back-translocate post-translocational ribosomes and increase the active fraction of newly translated proteins [32–34]. Its utility as a diagnostic marker has been previously demonstrated in a multiplex assay for the detection of the *Mycobacterium tuberculosis* complex (MTC) [35, 36].

In this study, we have developed and performed a preliminary evaluation of a real-time RPA assay based on the *LepA* gene for the detection of *S. pneumoniae*. We also developed a *LepA* based *S. pneumoniae* real-time PCR assay to compare in terms of specificity and sensitivity.

## Methods

### Bacterial strains and growth conditions

*S. pneumoniae* bacterial reference strains (*n* = 8), non-*S. pneumoniae* reference strains (*n* = 54) and invasive *S. pneumoniae* clinical isolates (*n* = 19) were evaluated in this study (Table 1). All strains were cultured overnight in brain heart infusion (BHI) medium (Oxoid, UK) at 37 °C. For enumeration, 100 μL of serially diluted bacterial suspensions were spread plated on Columbia blood agar and incubated for 24 hrs.

**Table 1:** Bacterial strains and reactivity in the RPA and PCR assays

Organism	Strain ID^a^	RPA	PCR
*S. pneumoniae*	DSM 20566	+	+
*S. pneumoniae*	DSM 11865	+	+
*S. pneumoniae*	DSM 11866	+	+
*S. pneumoniae*	DSM 14377	+	+
*S. pneumoniae*	DSM 24048	+	+
*S. pneumoniae*	DSM 11868	+	+
*S. pneumoniae*	DSM 25971	+	+
*S. pneumoniae*	DSM 14378	+	+
Clinical Isolates			
*S. pneumoniae*	Clinical Isolates (*n* = 19)	+	+
*S. agalactiae*	Clinical isolate (*n* = 1)	-	-
*N. meningitidis*	Clinical isolate (*n* = 1)	-	-
Non-*S. pneumonia* reference strains			
*S. agalactiae*	BCCM 15081	-	-
*S. agalactiae*	BCCM 15082	-	-
*S. agalactiae*	BCCM 15083	-	-
*S. agalactiae*	BCCM 15084	-	-
*S. agalactiae*	BCCM 15085	-	-
*S. agalactiae*	BCCM 15086	-	-
*S. agalactiae*	BCCM 15087	-	-
*S. agalactiae*	BCCM 15094	-	-
*S. agalactiae*	BCCM 15095	-	-
*S. anginosus*	BCCM 20563	-	-
*S. australis*	BCCM 15627	-	-
*S. bovis*	BCCM 20480	-	-
*S. canis*	BCCM 20715	-	-
*S. constellatus*	BCCM 20575	-	-
*S. cristatus*	DSM 8249	-	-
*S. downei*	DSM 5365	-	-
*S. dysgalactiae*	DSM 6176	-	-
*S. equi*	DSM 20561	-	-
*S. equinis*	DSM 20554	-	-
*S. gordonii*	DSM 6777	-	-
*S. infantis*	DSM 12492	-	-
*S. intermedius*	DSM 20573	-	-
*S. mitis*	DSM 12643	-	-
*S. mutans*	DSM 20523	-	-
*S. oralis*	DSM 20066	-	-
*S. parasanguinis*	DSM 6778	-	-
*S. perosis*	DSM 12493	-	-
*S. porcinus*	DSM 20725	-	-
*S. pseudopneumoniae*	DSM 18670	-	-
*S. pyogenes*	DSM 2072	-	-
*S. pyogenes*	DSM 20565	-	-
*S. salivarius*	DSM 20560	-	-
*S. salivarius*	DSM 20617	-	-
*S. sanguinis*	DSM 20567	-	-
*S. sinensis*	DSM 14990	-	-
*S. suis*	DSM 9682	-	-
*S. uberis*	DSM 20569	-	-
*S. vestibularis*	DSM 5636	-	-
*H. influenzae*	DSM 4690	-	-
*H. influenzae*	DSM 11121	-	-
*H. parainfluenzae*	DSM 8978	-	-
*H. haemolyticus*	CCUG 15312	-	-
*H. somnus*	CCUG 12839	-	-
*N. meningitidis*	DSM 10036	-	-
*K. pneumoniae*	DSM 30184	-	-
*P. aeruginosa*	DSM 50071	-	-
*E. coli*	DSM 30083	-	-
*E. faecalis*	DSM 20317	-	-
*C. albicans*	CBS 2700	-	-
*B. fragilis*	DSM 2151	-	-
*M. cattarhalis*	DSM 11994	-	-
*S. aureus*	DSM 346	-	-

^a^ *DSM* Leibniz-Institut, *DSMZ* Deutsche Sammlung von Mikroorganismen und Zellkulturen GmbH, *BCCM* Belgian Coordinated Collections of Microorganisms, *CCUG* Culture Collection, University of Göteborg, Sweden

### DNA extraction

Following overnight culture, bacterial genomic DNA was extracted and purified using the DNeasy Blood and Tissue kit (Qiagen, Germany) according to the manufacturers’ instructions. Following purification, DNA was quantified by fluorescence (Qubit DNA BR Assay, Life technologies, UK). Genome equivalents (GE) were calculated based on an assumed genome size of 2.1 Mb [37]. For whole blood spiking experiments, from an overnight culture, the culture medium was 10-fold serially diluted in BHI. 2 μL of the serially diluted bacterial suspensions, were spiked into 98 μL of fresh human whole blood. Subsequently, total genomic DNA was extracted and purified using the DNeasy Blood and Tissue kit (Qiagen, Germany) according to the manufacturers’ instructions, with the exception that DNA was eluted in 20 μL H_2_0 (rather than 200 μL buffer AE).

### Primer and probe design

*LepA* sequences were obtained from GenBank (http://www.ncbi.nlm.nih.gov/genbank), and the Functional Gene Pipeline and Repository (FunGene; http://fungene.cme.msu.edu) website. Sequences were aligned using the multiple sequence alignment tool ClustalW [38]. Following alignment, *S. pneumoniae* specific PCR and RPA primers and probes were manually designed (Table 2). The sequences were finally screened for homology using the BLASTn algorithm (http://blast.ncbi.nlm.nih.gov/Blast.cgi) to confirm their specificity. All oligonucleotide primers and probes were purchased from Integrated DNA Technologies (Leuven, Belgium), with the exception of the RPA probe, which was purchased from Biosearch Technologies (Petaluma, California, USA).

**Table 2:** Nucleic Acid sequences of primers and probes

Probe/primer	DNA sequence (5’-3’)	Nucleotide position^a^	Amplicon size (bp)
RPA			190
Forward	ACAGCTCCGTCTGTTATTTACAAAGTTAATTTGA*C	1123–1157	
Reverse	AGTCCCCACGCTTACGCTGAGCTAGCTCCATTAC*T	1278–1312	
Probe	CTTGACATAAGGCTCTTCAATGGTCGCAATCT[T(FAM)]A(dSpacer)[T(BHQ-1)]TGGGTCTGGAAAC*T	1193–1242	
PCR			107
Forward	CTCGTAAGCGTAAACTCCTTG	1706–1727	
Reverse	CATACTCAAGACGCTGAGGA	1793–1813	
Probe	FAM-ACGCATGAAATCCATCGGATCAGTT-TAMRA	1749–1773	

^a^The nucleotide position refers to the LepA gene from *S. pneumoniae* strain SPNA45 (Genbank accession number NC_018594.1)

*indicates a phosphorothioate bond

### Real-time recombinase polymerase amplification

RPA reactions were performed using the TwistAmp Exo® kit (TwistDx, Cambridge, UK) and contained: 1 x rehydration buffer, 4 μL forward and reverse primer (6 μM each), 4 μL probe (6 μM) and 1 μL genomic DNA as the amplification template. To initiate the reaction, 14 mM magnesium acetate (2.5 μL at a concentration of 280 mM) was added. Reactions were performed in a total volume of 50 μL at 40 °C in a portable real-time fluorometer (Twista®, TwistDx) for 20 minutes with a mixing and centrifugation step after the first 4 minutes. External negative and positive amplification controls were included in each run. The positive amplification control consisted of 100 GE of the *S. pneumoniae* type strain (DSM 20566) diluted in molecular grade water. The no-template control (NTC) consisted of molecular grade water.

### Real-time PCR

All real-time PCR assays were performed using a LightCycler® 480 real-time PCR instrument (Roche Diagnostics, UK). PCR reactions were performed in 200 μL 12-well optical strips (BIOplastics BV, The Netherlands) in a total volume of 20 μL. Each reaction consisted of 1 x LightCycler Probes Master mix (Roche Diagnostics, UK), 10pmoles each primer and 5pmoles of probe and 1 μL genomic DNA as the amplification template. Negative and positive amplification controls were included in each run. The positive amplification control consisted of 100 GE of the *S. pneumoniae* type strain (DSM 20566) diluted in molecular grade water. The no template control (NTC) consisted of molecular grade water. The cycling parameters consisted of 1 cycle of 95 °C for 5mins followed by 45 cycles of 95 °C for 10s, 60 °C for 15 s and 72 °C for 1 s.

### Specificity analysis/determination

The specificity of both the RPA and PCR assays were evaluated. Genomic DNA (5 × 10^4^ GE) from a panel of *Streptococcus* and non-*Streptococcus* organisms (see Table 1) was tested in both RPA and PCR amplification assays.

### Sensitivity analysis/determination

The analytical limit of detection (95 % confidence level) of the PCR and RPA assays were determined. *S. pneumoniae* (DSM 20566) genomic DNA was diluted in molecular grade water to 8, 7, 6, 5, 4, 3, 2, or 1 GE per microliter. PCR and RPA reactions were then performed using 1 μL of the diluted DNA as the amplification template. Reactions were performed in replicates of twelve and the subsequent data was analysed statistically (Probit, Minitab, Version 16).

### Evaluation of inhibition of RPA by human DNA

In order to determine the limit of background human DNA tolerated by the RPA and PCR reactions, varying quantities of human genomic DNA (100, 200 and 400 ng) were added to reactions containing 50, 20 or 4 *S. pneumoniae* (DSM 20566) GE.

### Blood spiking experiments

Human whole blood was collected from a healthy volunteer in EDTA vacutainers (Becton Dickinson and Company, Ireland). Two microliters of 10-fold serially diluted bacterial suspensions (over five orders of magnitude) were spiked into 98 μL of fresh (within 1 hr of sampling) human whole blood. Aliquots (100 μL) of the serially diluted bacterial suspensions were plated on blood agar for enumeration. The spiked blood samples were then processed immediately to extract DNA. From the eluted DNA (20 μL), 1 μL was then subjected to RPA or PCR amplification.

### Clinical sample analysis using RPA and PCR

Whole blood was collected in EDTA blood tubes from patients attending Galway University Hospital, suspected of a bloodstream infection for routine full blood count and from healthy volunteers. Generally, within 24 hours of collection of patient blood, residual material was collected, anonymised and then stored at −80 °C. Blood collected from the healthy volunteers was also stored at −80 °C. Subsequently, the samples were thawed on ice and total genomic DNA was extracted from 100 μL of sample and eluted in 20 μL molecular grade water as described previously. For analysis, 1 μL or 5 μL of the eluted genomic DNA was subjected to amplification by RPA and PCR.

### Ethical approval

Ethical approval for this study was granted by the Ethics Committee of the Galway University Hospital, Galway, Ireland. Participants were selected on the basis of having blood cultures submitted to the Department of Medical Microbiology for detection of blood stream infection. Following full blood count, patients for whom residual EDTA blood samples were available were recruited into the study based on the results of the blood culture (confirmed positive for the presence of *S. pneumoniae*). For controls, blood was collected from healthy individuals, following informed consent.

## Results

### Analytical specificity

**Fig. 1:**
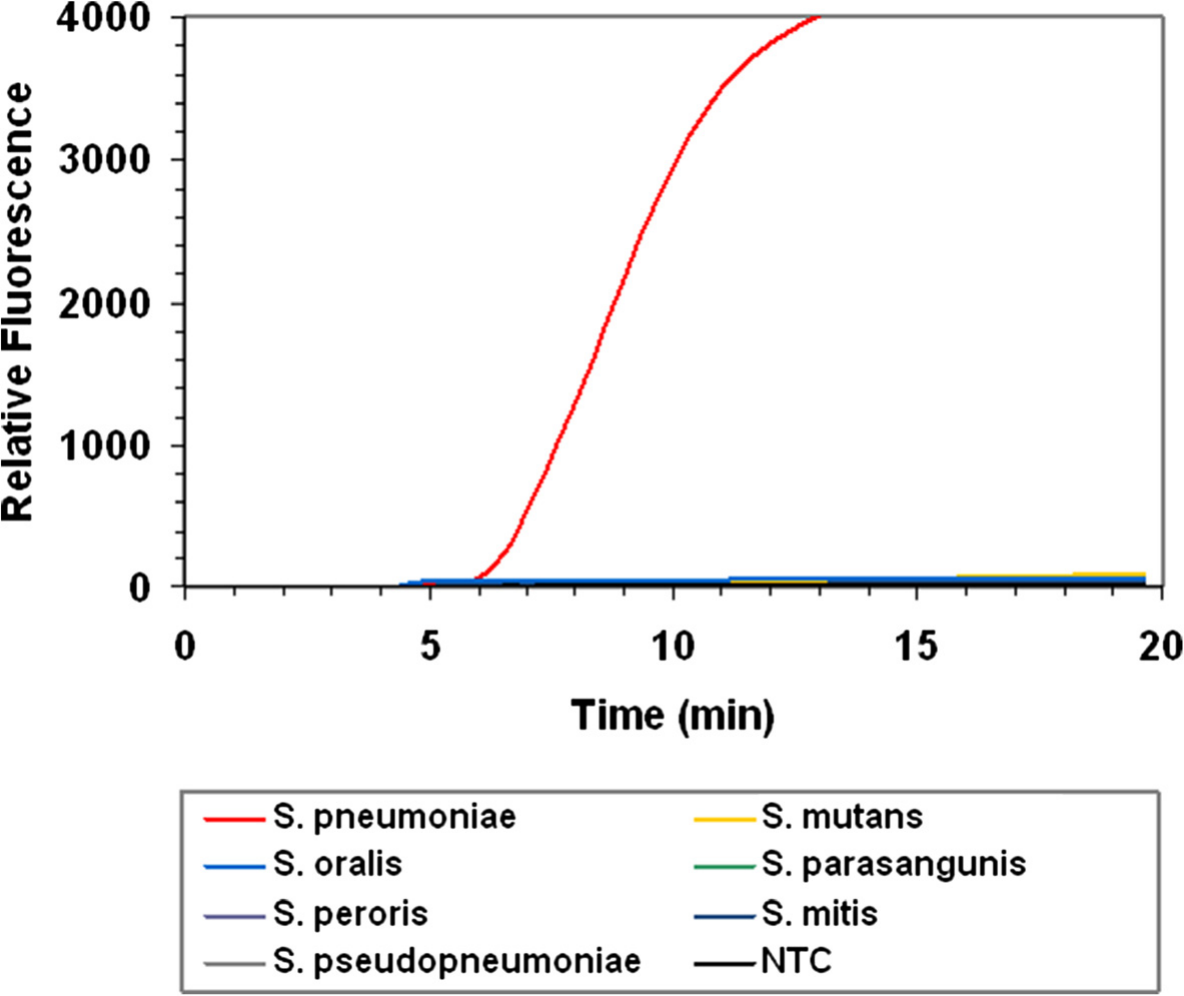
RPA assay specificity. The graph shows the fluorescence intensity of an assay containing 100 genome equivalents (GE) of *S. pneumoniae* (type strain; DSM20566), 5 x 10^4^ GE of 6 streptococcus species and a no template control (NTC)

The analytical specificities of both the RPA and real-time PCR assays were determined and are shown in Table 1. Both assays detected the 3 *S. pneumoniae* references strains tested. Both assays also detected all invasive clinical isolates (*n* = 19). Of the 39 other *Streptococcus* and non-*Streptococcus* species that were tested, none were detected by either of the assays, indicating the analytical specificity of the assays (Table 1, Fig. 1).

### Analytical limit of detection

**Fig. 2:**
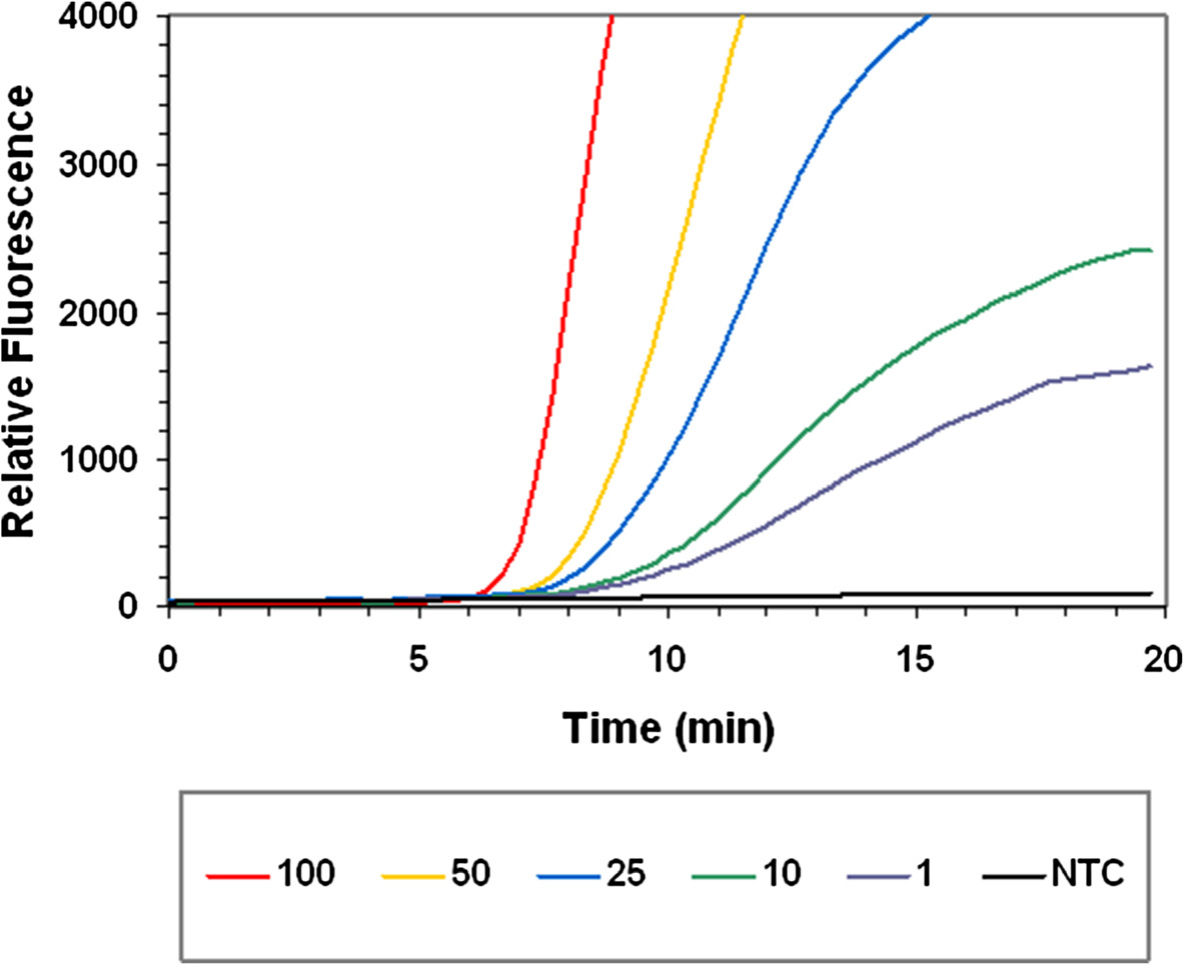
RPA assay sensitivity. The graph shows the fluorescence intensity obtained from the amplification of 100, 50, 25, 10 and 1 genome equivalents of *S. pneumoniae* (type strain; DSM20566). A no template control (NTC) reaction is also shown

Genomic DNA was serially diluted (100–1 GE per reaction) in water and subjected to RPA. Initial experiments suggested that the RPA was capable of detecting 1 GE per reaction (Fig. 2). Subsequently, the analytical limit of detection of both the RPA and real-time PCR assays were measured by testing 12 replicates of serially diluted genomic DNA from the *S. pneumoniae* type strain (DSM 20566). The analytical limit of detection (95 % confidence level) of the RPA and PCR assays was established to be 4.1 and 5.1 GE per reaction, respectively. Table 3 outlines the number of reactions producing a positive result for each template quantity.

**Table 3:** Analytical sensitivity

Input Level	# of Replicates Tested	# of Replicates Detected	# of Replicates Detected
Stress (GE)		RPA	PCR
8	12	12	12
7	12	12	12
6	12	12	12
5	12	12	11
4	12	12	11
3	12	9	8
2	12	8	9
1	12	8	4

The number of replicates producing positive signals following RPA and PCR amplification of varying (8, 7, 6, 5, 4, 3, 2, or 1) *S. pneumoniae* genome equivalents (GE)

### Assessment of inhibition and detection in whole blood

**Fig. 3:**
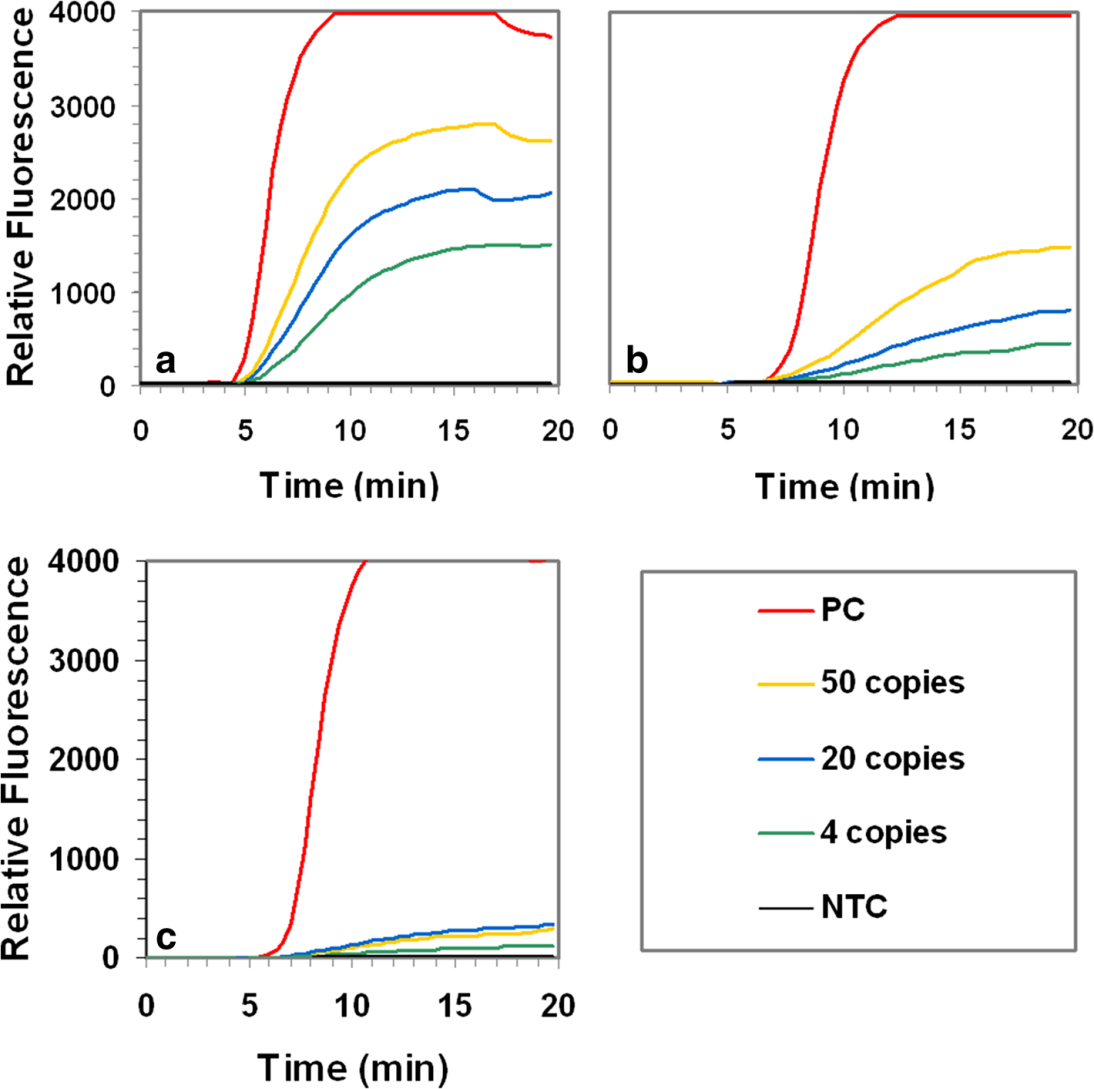
RPA assay robustness. The graphs show the fluorescence intensity obtained from the amplification of 50, 20 and 4 genome equivalents (GE) of *S. pneumoniae* (type strain; DSM20566), **a** in the absence of background human genomic DNA, **b** in the presence of 200 ng background human genomic DNA and **c** in the presence of 400 ng background human genomic DNA. Also shown in each graph is the fluorescence intensity obtained from the amplification of 100 GE (positive control, PC) of *S. pneumoniae* (type strain; DSM20566) and a no template control reaction (NTC)

The degree to which the RPA reaction was inhibited by the presence of human DNA was concentration dependant, varying from no significant inhibition (100 ng; data not shown) to substantial inhibition in the presence of 400 ng human DNA (the highest tested). Whilst inhibited, when spiked with 200 ng or 400 ng of human DNA, 50, 20 and 4 *S. pneumoniae* GE were detected, all producing signals above that of the negative control reaction (Fig. 3). When the PCR reaction was spiked with human DNA (100, 200 or 400 ng/reaction), there was no discernible shift in threshold-cycle between spiked and non-spiked reactions, indicating that the PCR was not inhibited by the presence of human DNA (data not shown).

**Fig. 4:**
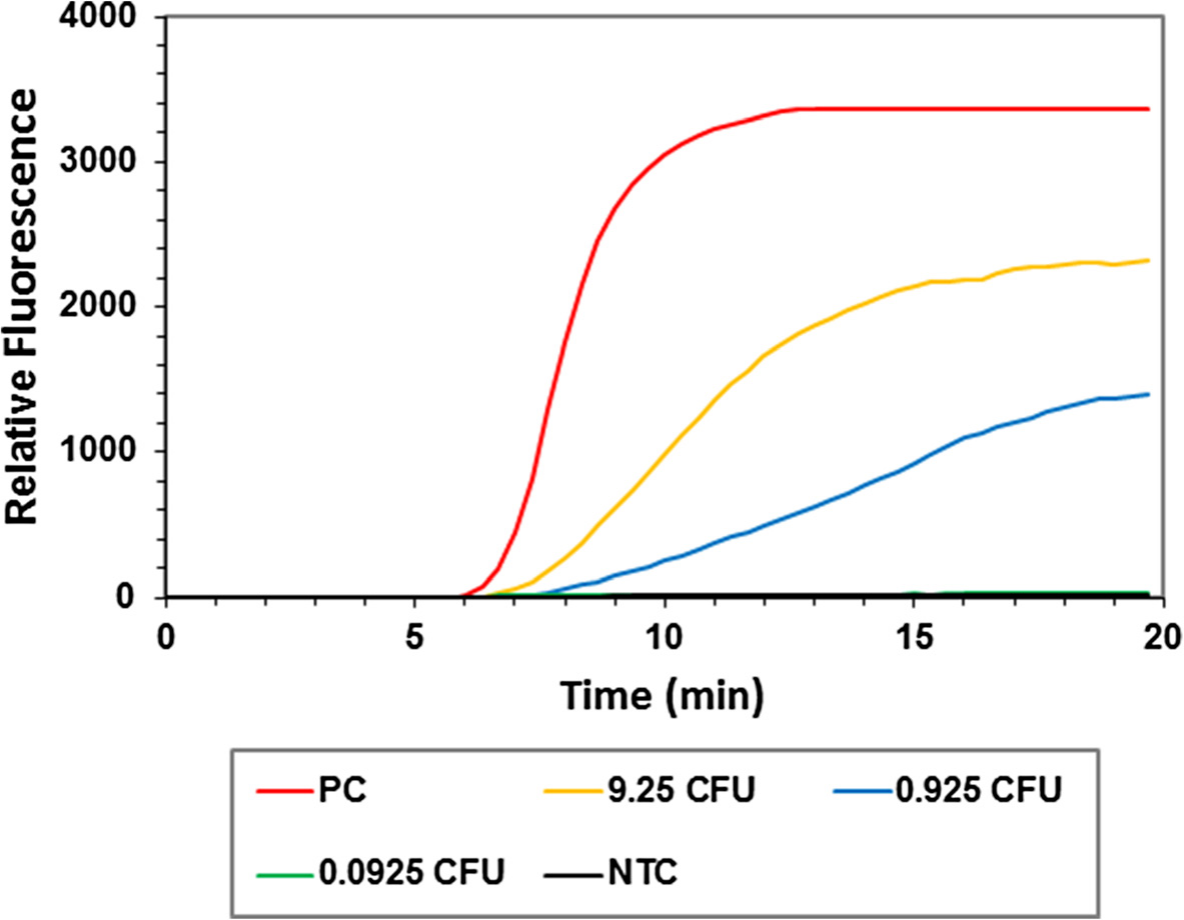
Blood spiking. The graph shows the fluorescence intensity obtained from the RPA of 1 μL purified DNA (9.25, 0.925 and 0.0925 CFU equivalents) extracted from whole blood spiked with varying CFU of *S. pneumoniae* (type strain; DSM20566). Also shown is the fluorescence intensity obtained from the amplification of 100 GE (positive control, PC) of *S. pneumoniae* (type strain; DSM20566) and a no template control reaction (NTC)

The ability of the assays to detect *S. pneumoniae* spiked into whole blood from a healthy volunteer was also determined. Plate counts indicated that 0.0185, 0.185, 1.85, 18.5, 185 CFU had been spiked into 98 μL of blood. Upon extraction, total DNA was eluted into 20 μL, of which 1 μL was subjected to RPA and PCR, meaning that each reaction contained template genomic DNA from 0.000925, 0.00925, 0.0925, 0.925 or 9.25 CFU equivalents. Both the RPA and PCR assays detected 0.925 and 9.25 CFU equivalents per reaction (Fig. 4). The lower quantities (0.000925, 0.00925, 0.0925 CFU) were not detected.

### Detection of *S. pneumoniae* in clinical samples

**Fig. 5:**
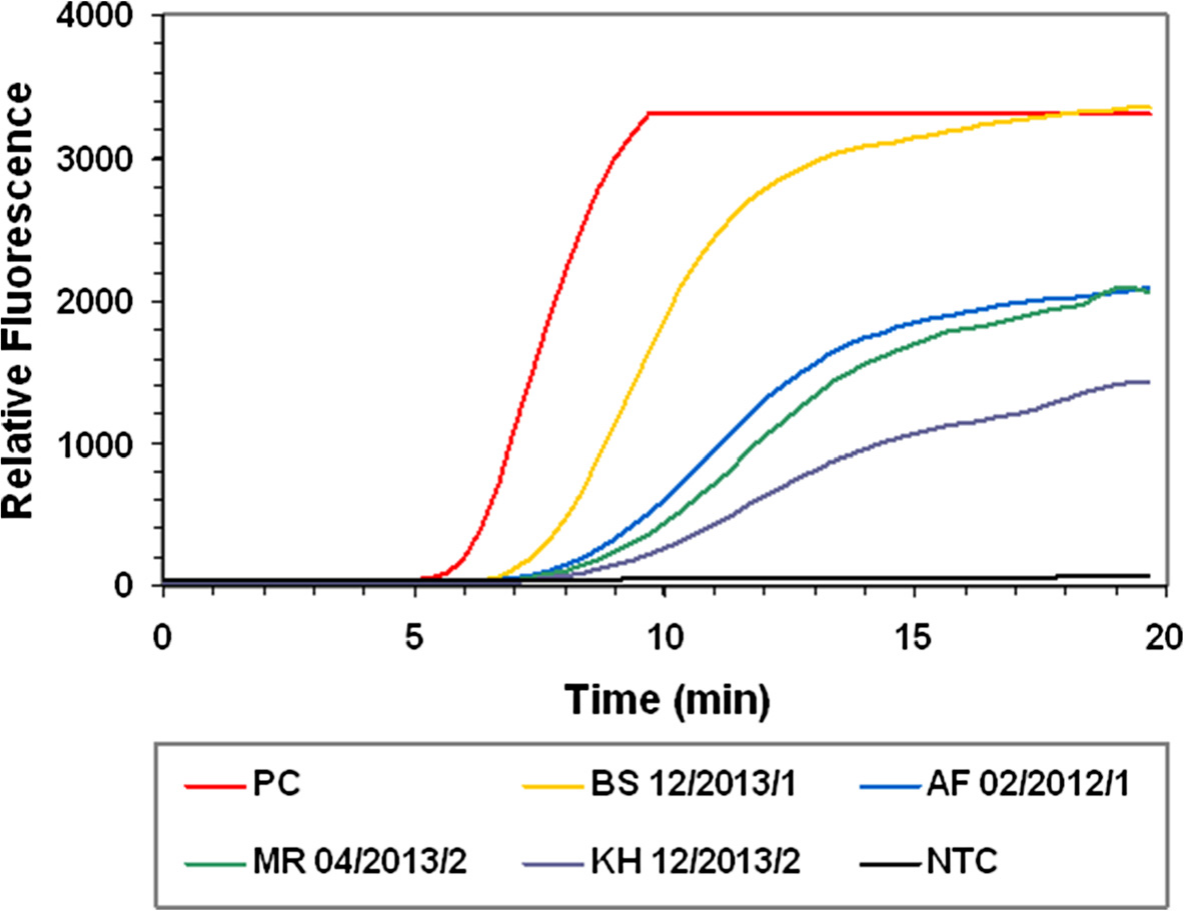
Analysis of clinical samples. The graph shows the fluorescence intensity from the amplification of extracted genomic DNA from 4 clinical samples previously confirmed positive for *S. pneumoniae* by culture. Also shown in each chart is the fluorescence intensity obtained from the amplification of 100 GE and a no template control reaction (NTC)

Eleven blood samples confirmed by culture to be positive for *S. pneumoniae* were subjected to analysis by RPA and PCR. Both the RPA and PCR assays were positive for the same 8 of the 11 samples tested (Table 4). For the remaining 3 *S. pneumoniae* positive samples, 5 μL of the purified DNA was subsequently tested in the RPA and PCR assays, resulting in positive signals. Figure 5 shows representative RPA amplification curves from the resultant DNA purified from 4 confirmed positive clinical blood samples. RPA and PCR analysis of DNA extracted from the blood of four healthy (culture negative) volunteers, produced negative results.

**Table 4:** Analysis of clinical samples. RPA and PCR assay reactivity

Sample ID^a^	RPA / PCR^b^	
	1 μL	5 μL
BS 12/2013/1	+	
BS 12/2013/2	+	
KH 12/2013/1	-	+
KH 12/2013/2	+	
DK 10/2011/2	+	
MG 03/2012	+	
EM 03/2012	-	+
MF 03/2012	-	+
AF 02/2012/1	+	
AF 02/2012/2	+	
MR 04/2013/2	+	
HC1		-
HC2		-
HC3		-
HC4		-

^a^Samples HC1, HC2, HC3 and HC4 were obtained from healthy individuals

^b^1 or 5 μL of purified DNA was required to produce a positive signal in the RPA or PCR

## Discussion

Some 93 polysaccharide capsular types or serotypes of *S. pneumoniae* have been identified [39]. The introduction of a 7-, 13-, and more recently, a 23-valent conjugate vaccine has been associated with a dramatic reduction in the number of deaths, particularly amongst children and in adults (via a herd effect) due to pneumococcal infection [40, 41]. However, pneumococcal disease remains a serious disease globally, particularly in children less than two years old, the elderly (>65 years) and in immunocompromised individuals. Thus, a robust diagnostic assay capable of the rapid, sensitive and specific detection of *S. pneumoniae* in near-patient settings could play a significant role in the reduction of pneumococcal disease related morbidity and mortality, particularly in developing countries.

Isothermal *in vitro* nucleic acid amplification strategies are of great interest in molecular diagnostics, as they negate the need for thermocycling, as is the case for PCR. In PCR, thermocycling is required to facilitate the separation of double-stranded DNA to enable amplification. The major isothermal amplification techniques currently utilised include: Nucleic Acid Sequence based Amplification (NASBA) [42], Transcription Mediated Amplification (TMA) [43], Loop-Mediated Isothermal Amplification (LAMP) [44], Helicase Dependant Amplification (HDA) [45], Rolling Circle Amplification (RCA) [46] and Strand Displacement Amplification (SDA) [47]. Of these, NASBA, TMA, RCA and SDA cannot be considered truly isothermal as they require an initial heating step to denature the target nucleic acid prior to amplification. RPA, HDA and LAMP can be considered truly isothermal, as there is no requirement for a denaturation step to initiate amplification. The operating temperature of LAMP is typically 60–65 °C, at which temperature the nucleic acid target is in dynamic equilibrium, enabling the binding of primers to their target sequence and subsequent amplification. Whilst LAMP is inexpensive to perform, primer design is complex and multiplex amplification is at present difficult. Both HDA and RPA use proteins to facilitate the binding of primers to their respective template and both technologies enable multiplex amplification. The major advantage of RPA over other competing isothermal amplification technologies is its speed, being capable of amplifying single-digit copy numbers of nucleic acids to detectable levels in 5–10 minutes.

In this study, we have developed real-time RPA and real-time PCR assays for the detection of *S. pneumoniae* using primers and probes targeting the *LepA* gene. We examined the ability of both assays to detect *S. pneumoniae* spiked into human whole blood. Finally, we applied both assays to the detection of *S. pneumoniae* directly in the blood of 11 patients confirmed positive for infection by blood culture.

Both RPA and PCR assays were highly specific, with all laboratory strains (*n* = 8) and clinical isolates of *S. pneumoniae* (*n* = 19) positively identified. Both assays were negative for all non-*S. pneumoniae* organisms (*n* = 39) assayed. These data support the results of our *in silico* analysis, demonstrating that the *LepA* gene has sufficient sequence heterogeneity to enable the specific detection of *S. pneumoniae*. Probit regression analysis was used to calculate (with 95 % confidence) the analytical LOD of both assays. The RPA assay had a marginally better LOD than the PCR assay (4.0 and 5.1 GE respectively). Assuming Poisson distribution, both assays demonstrated limits of detection approaching 3 molecules, the most sensitive LOD theoretically possible [48]. It should also be noted that the RPA reactions were performed in volumes of 50 μL, 250 % greater than the 20 μL PCR reactions, suggesting that by reducing the reaction volume and thereby increasing the effective concentration of the initial template DNA, the assay sensitivity could be further improved.

RPA is a rapid isothermal *in vitro* nucleic acid amplification technology. When 10 *S. pneumoniae* GE were used as the template for amplification, our RPA assay produced a positive signal in as little as 5 mins. This is in contrast to 35 cycles (approximately 20mins), which was required to produce a positive signal in our PCR assay for the equivalent template quantity.

Prior to performing our spiking experiments we investigated what affect the presence of background human DNA had on the RPA assay performance. We found that in the presence of 400 ng background DNA (the highest quantity tested), although considerably inhibited, the RPA assay was capable of detecting 50, 20 and 4 GE, all giving signals above the no template control reaction. In the presence of 200 ng of background human DNA, the RPA assay was also inhibited, but to a much lesser degree than in the presence of 400 ng DNA. According to the DNA extraction kit manufacturer (Qiagen), 3–6 μg is the typical yield of genomic DNA obtained from 100 μL of mammalian blood. The DNA we used for the inhibition studies was extracted using the DNeasy blood and tissue kit and yields were in this range (data not shown). Assuming the average yield is 4.5 μg DNA /100 μL blood, and considering that we eluted our extracted DNA into 20 μL, of which 1 μL is subjected to RPA, the quantity of background human DNA in the RPA reaction is approximately 225 ng. We also tested the effect of background human DNA on the PCR assay and found that the reaction was not inhibited, even in the presence of 400 ng of background DNA.

When spiked into whole blood, both PCR and RPA assays were capable of detecting 0.925 CFU/reaction, which equals 18.5 CFU equivalents/100 μL blood. Reactions in the blood spiking experiment were performed in duplicate and subsequently repeated to verify this result. Peter *et al.* (2009) developed a real-time PCR assay targeting the autolysin gene capable of detecting the equivalent of 125 CFU in 1 mL whole blood [49]. It should be noted, that CFU is an estimate of the number of viable organisms present. CFU as a metric does not account for the presence of dead, non-viable cells or for organisms that tend to form clumps or grow as pairs or chains and may well substantially underestimate the number of living cells in a sample [50].

To further evaluate our RPA assay, we tested a small number of *S. pneumoniae* culture positive (*n* = 11) and culture negative (obtained from consenting volunteers) blood samples (*n* = 4). All culture positive samples resulted in positive signals when analysed by RPA and PCR amplifications while the four culture negative samples were RPA and PCR negative. Eight of the 11 culture positive samples produced positive signals for RPA and PCR when tested with 1 μL of purified DNA. The remaining three that tested negative using 1 μL of purified DNA, were then tested using 5 μL of purified DNA in both assays, with both assays producing positive signals. This result hints at a reduced bacterial DNA load or the presence of RPA and PCR reaction inhibitors in the 3 of the 11 culture positive samples in question. This may have been the result of lower bacterial cell load in the samples, DNA degradation due to extended sample processing time during sample acquisition.

## Conclusions

We have developed a sensitive and specific real-time RPA and a real-time PCR assay for the detection of *S. pneumoniae* in clinical blood samples. We have shown this assay to be as specific and as, sensitive as PCR when applied to the detection of *S. pneumoniae* using the *LepA* gene as a diagnostics target. This assay may find utility as a rapid point-of-care diagnostic test for the detection of *S. pneumoniae*. Future work will focus on the development of an internal amplification control for the assay and increasing the number of clinical samples to fully validate the RPA assay.
